# Supplementary material for: Cotton plant defence against a fungal pathogen is enhanced by expanding BLADE-ON-PETIOLE1 expression beyond lateral-organ boundaries
Source: Commun Biol. 2019 Jun 21;2:238. doi: 10.1038/s42003-019-0468-5 (PMC6588604; doi:10.1038/s42003-019-0468-5)
Supplement: Supplementary file 1 — Supplementary Information [file 42003_2019_468_MOESM1_ESM.pdf]

Supplementary Information

Supplementary Figure 1

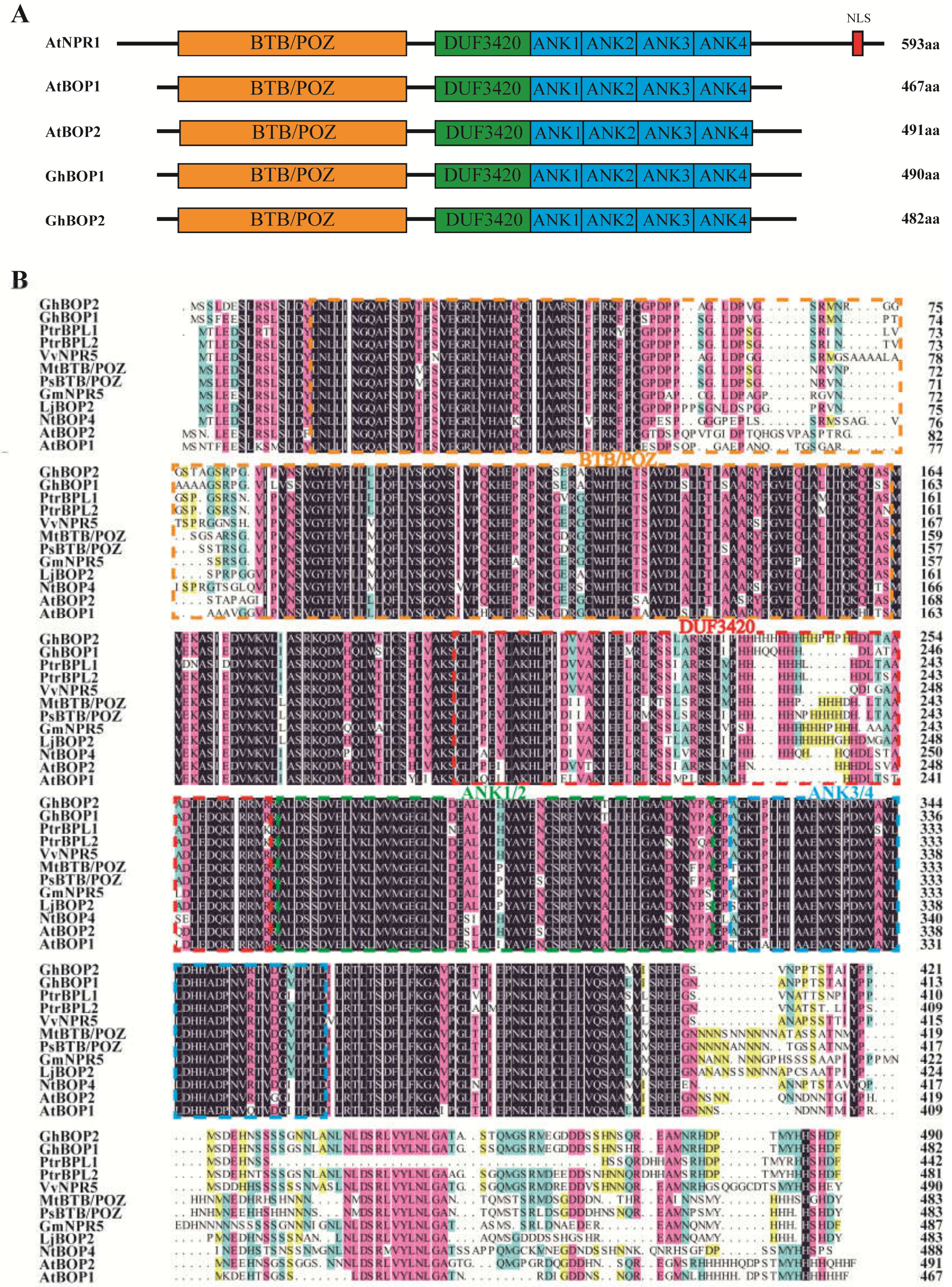

Supplementary Figure 1. Domain structures of BOPs and NPR1 and amino acid sequence analysis of BOPs from different plant species.

(A) Structural drawing of BOPs from *G. hirsutum* and *A. thalaisana* compared with AtNPR1. BTB/POZ domain (Pfam: 00651), DUF3420 domain (Pfam: 11900), Blue, ANK domain

(Pfam: 00023) and NLS (Nuclear localization signal) were indicated in orange, green, blue and red, respectively.

(B) Amino acid alignment of BOP proteins from *G. hirsutum* and other species. The complete amino acid sequences of BOPs were acquired from NCBI database and performed on DNAMAN 7.0. The conserved domain of BTB/POZ, DUF3420, ANK1/2 and ANK3/4 was noted in dotted line frame with orange, red, green and blue, respectively.

Supplementary Figure 2

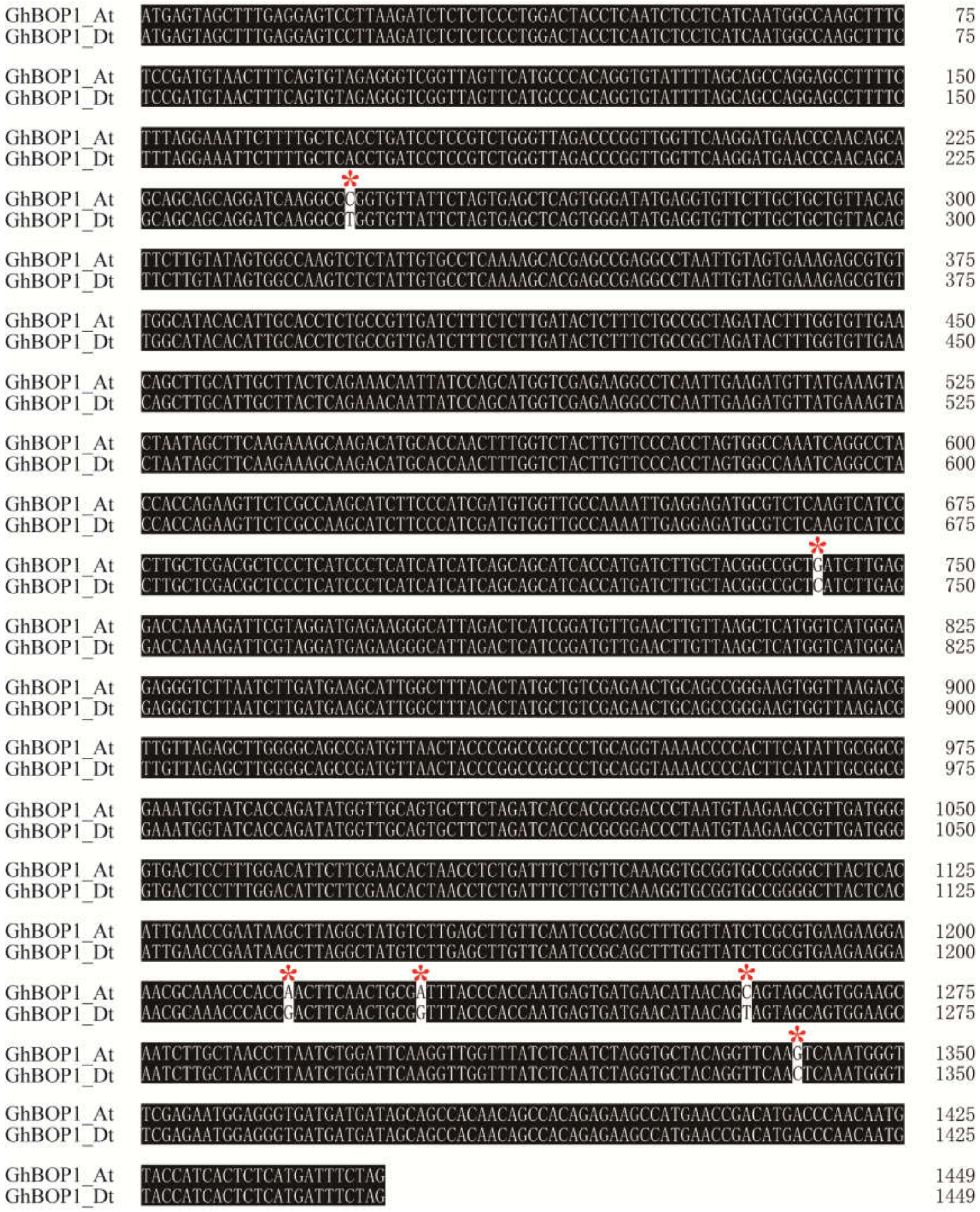

Supplementary Figure 2. Alignment of CDS sequences between *GhBOP1* at At and Dt subgenome. The red asterisks indicate the mismatched nucleotides.

Supplementary Figure 3

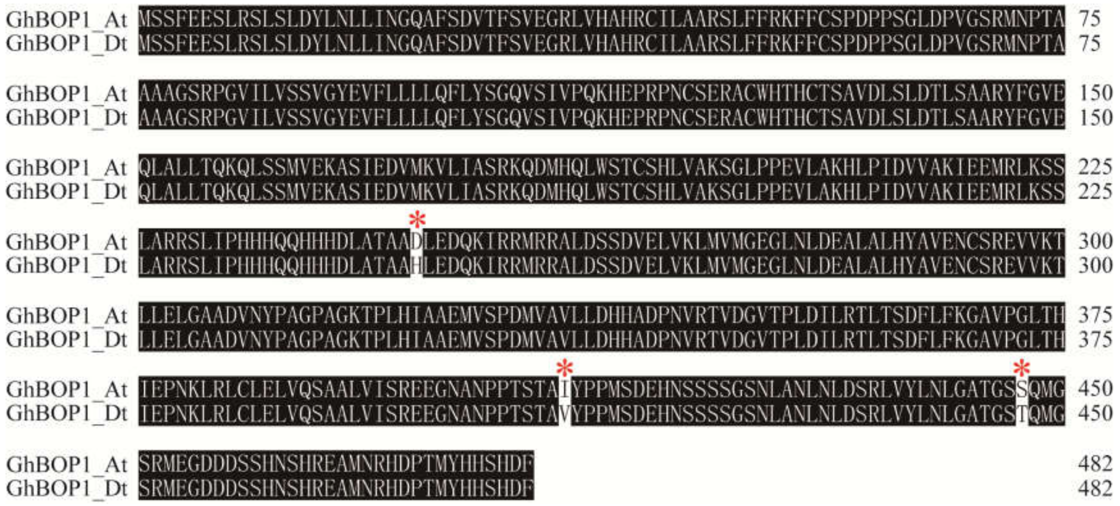

Supplementary Figure 3. Alignment of amino acid sequences between *GhBOP1* at At and Dt subgenome. The red asterisks indicate the mismatched amino acids.

Supplementary Figure 4

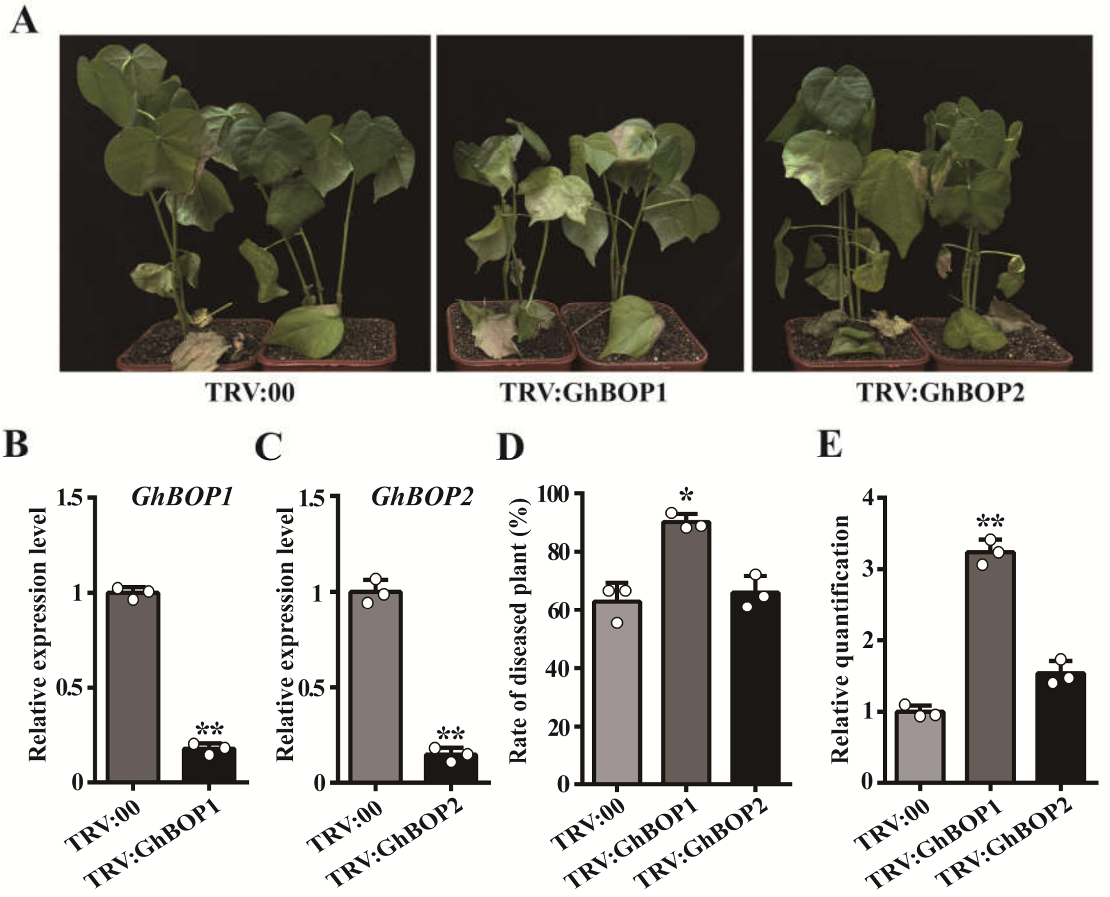

Supplementary Figure 4. *GhBOP1*-silenced plants decreased the resistance to *V. dahliae*.
(A) Disease symptoms of TRV:00, TRV:GhBOP1 and TRV:GhBOP2 plants inoculated with *V. dahliae*.
(B) Relative expression levels of *GhBOP1* in roots of TRV:00 and TRV:GhBOP1 plants. Error bars represent the SD of three biological replicates. Asterisks indicate statistically significant differences, as determined by Student's *t*-test (**$P < 0.01$).
(C) The relative expression levels of *GhBOP2* in roots of TRV:00 and TRV:GhBOP2 plants. Error bars represent the SD of three biological replicates. Asterisks indicate statistically significant differences, as determined by Student's *t*-test (**$P < 0.01$).
(D) Diseased rate analysis of TRV:00, TRV:GBOP1 and TRV:GhBOP2 plants. Error bars represent the SD of three biological replicates. Asterisks indicate statistically significant differences compared to TRV:00 as determined by Student's *t*-test (**$P < 0.01$).
(E) Relative quantification of the fungal biomass in the infected stems of TRV:00, TRV:GBOP1 and TRV:GhBOP2 plants. Error bars represent the SD of three biological replicates. Asterisks indicate statistically significant differences compared to TRV:00, as determined by Student's *t*-test (**$P < 0.01$).

Supplementary Figure 5

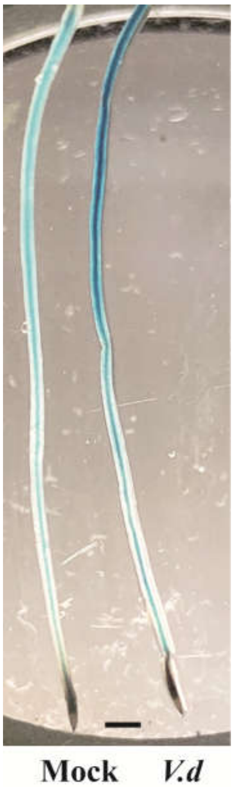

Supplementary Figure 5. GUS staining analysis the expression level of GhBOP1. The expression level of GhBOP1in the roots of GhBOP1pro:GUS transgenic plants were analyzed by GUS staining at 3 d after inoculated with *V. dahlia*e. The scale bars indicate 1 mm.

Supplementary Figure 6

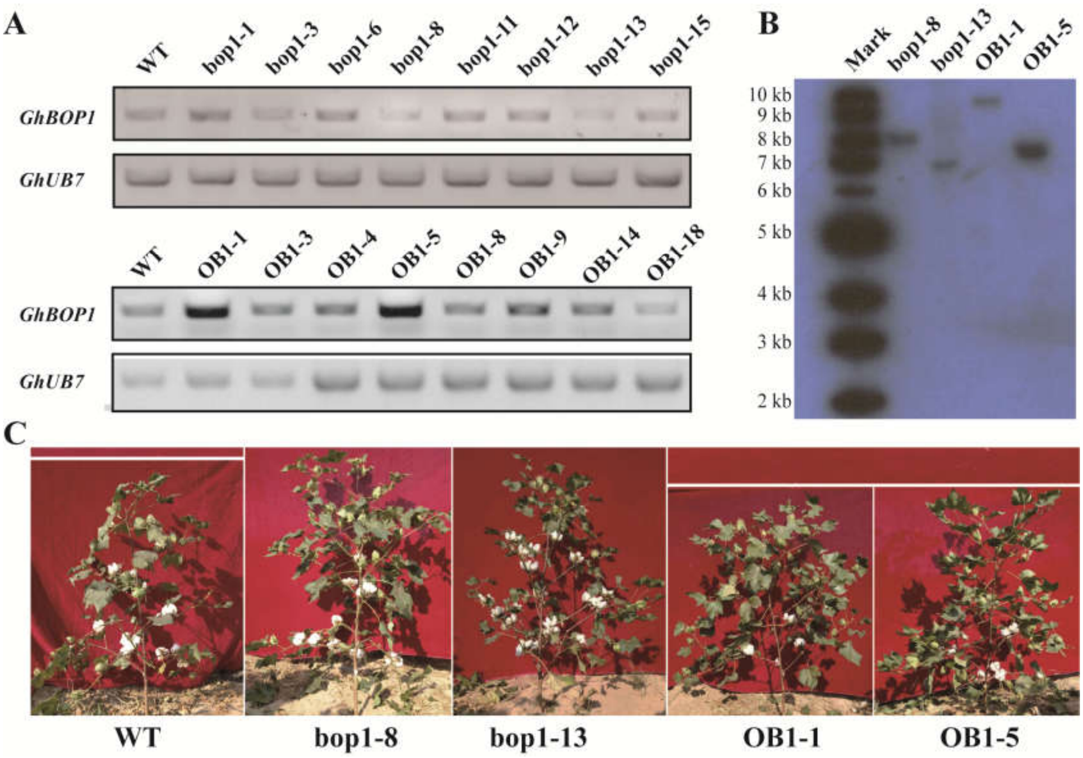

Supplementary Figure 6. Molecular identification and adult phenotypes of GhBOP1 transgenic plants.

(A) PCR was performed to detect the *GhBOP1* and the *GhUB7* expression levels in roots. The *GhUB7* gene was used as internal control.

(B) Southern blotting analysis in the four transgenic lines.

(C) Adult phenotypes of WT and transgenic cotton plants.

Supplementary Figure 7

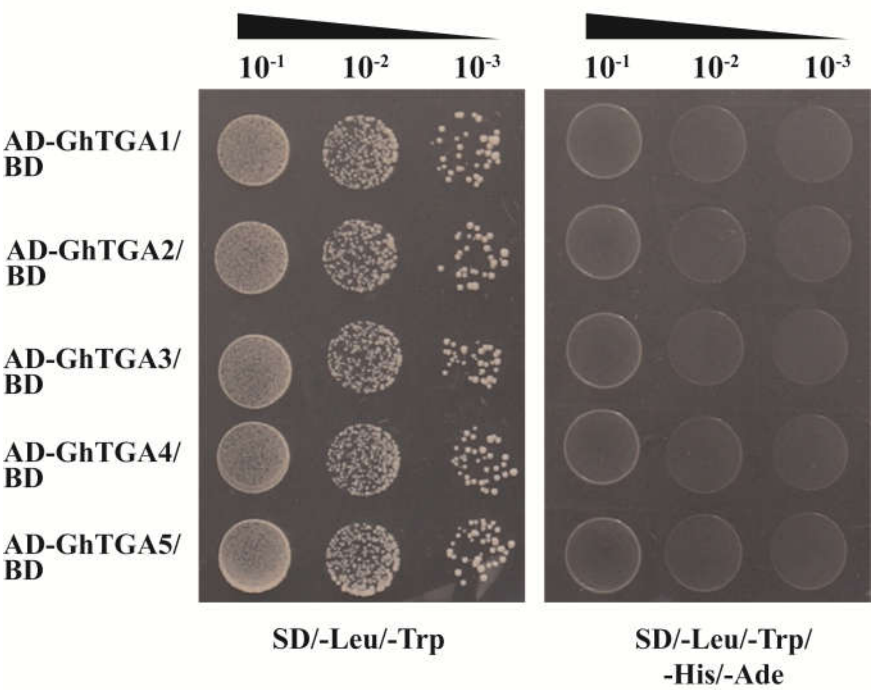

Supplementary Figure 7. Self-activation assay of AD-GhTGAs in yeast.

Supplementary Figure 8

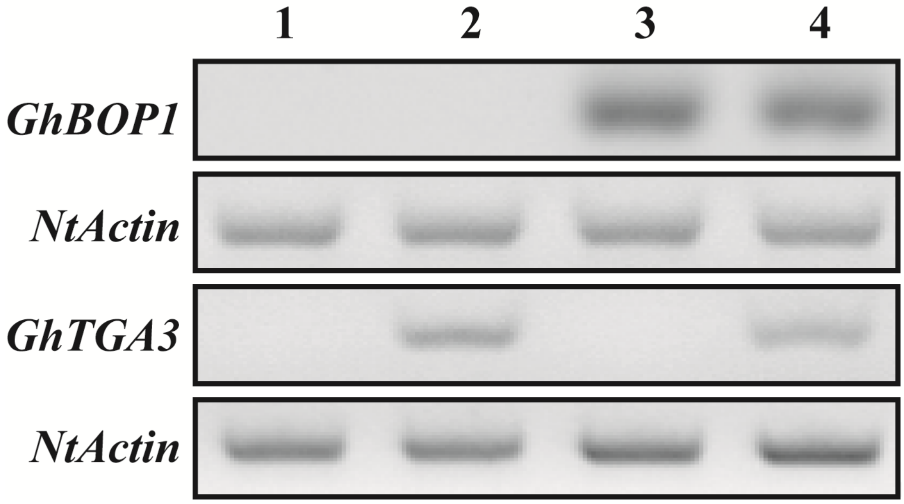

Supplementary Figure 8. RT-PCR detected the *GhBOP1* and *GhTGA3* expression levels in tobacco leaf at 48h after infiltrated by the vectors shown in Figure 6A.

Supplementary Figure 9

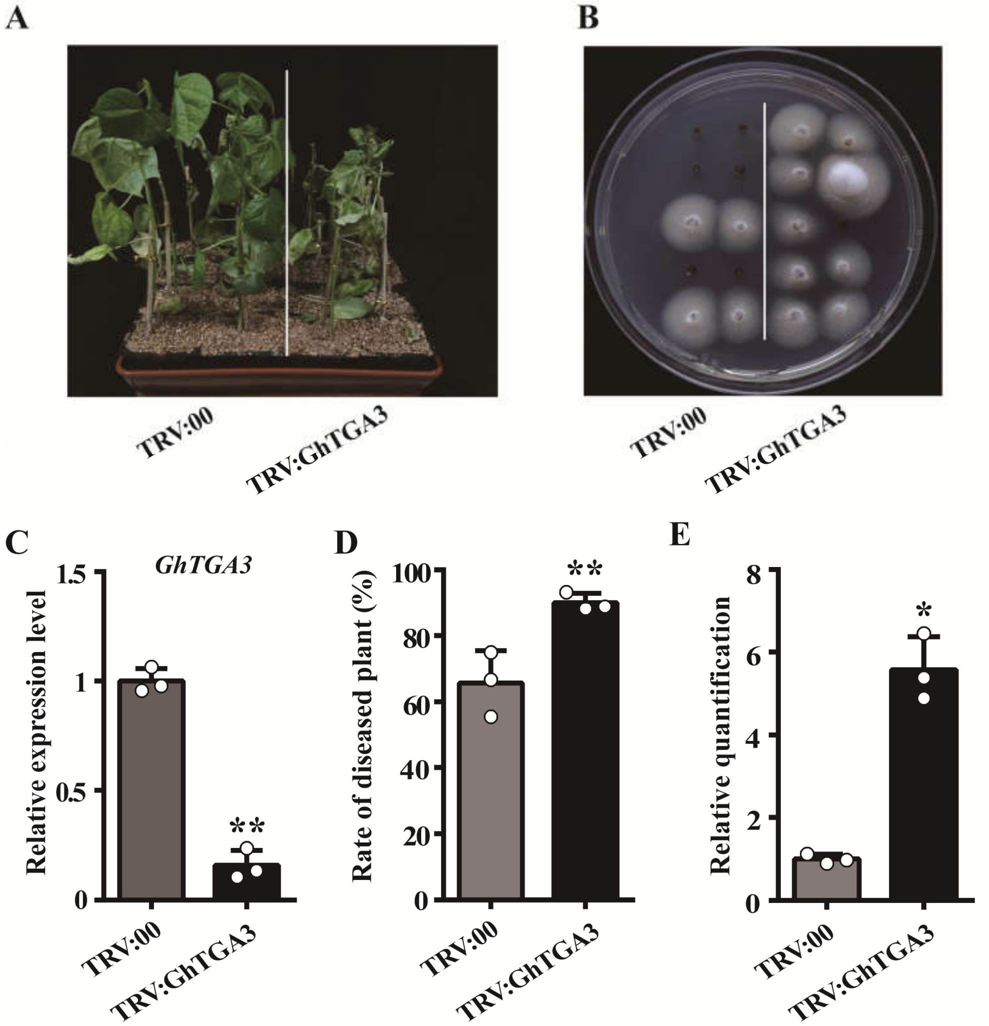

Supplementary Figure 9. GhTGA3 positively regulating plant defence against *V. dahliae*

(A) Disease symptoms of TRV:00 and TRV:GhTGA3 inoculated with *V. dahliae*. Photographs were taken at 18 d after pathogen inoculation.

(B) Fungal recovery assay to detect the morbidity of TRV:00 and TRV:GhTGA3 plants. The stem section of the inoculated plants was placed on PDA medium and taken photographs at 4 d after culture.

(C) Relative expression levels of *GhTGA3* in roots of TRV:00 and TRV:GhTGA3 plants. Error bars represent the SD of three biological replicates. Asterisks indicate statistically significant differences, as determined by Student's *t*-test (***P* < 0.01).

(D) Diseased rate analysis of TRV:00 and TRV:GhTGA3 plants. Error bars represent the SD of three biological replicates. Asterisks indicate statistically significant differences compared to TRV:00 as determined by Student's *t*-test (***P* < 0.01).

(E) Relative quantification of the fungal biomass in the infected stems of TRV:00 and TRV:GhTGA3 plants. Error bars represent the SD of three biological replicates. Asterisks indicate statistically significant differences, as determined by Student's *t*-test (**P* < 0.05).

Supplementary Figure 10

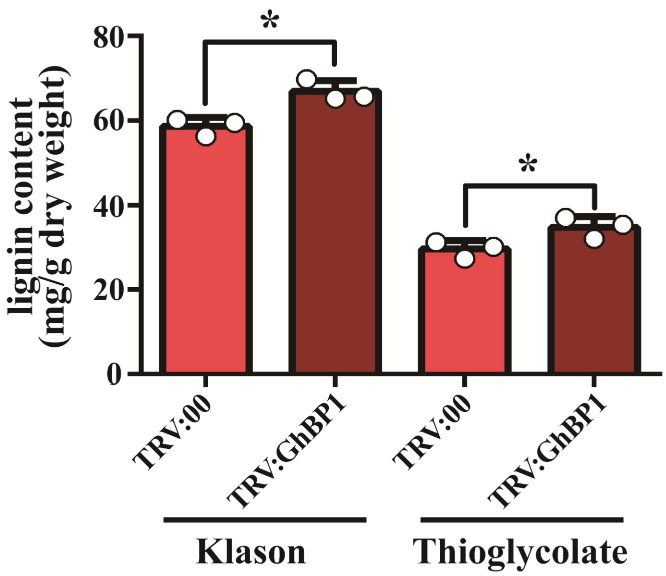

Supplementary Figure 10. GhBP1 negatively regulating lignin deposition. Analysis of lignin content in *GhBP1*-silenced plant stems through Klason and thioglycolate methods. Error bars represent the SD (n=18) of three biological replicates. Asterisks indicate statistically significant differences, as determined by Student's *t*-test (**$P < 0.01$).

Supplementary Figure 11

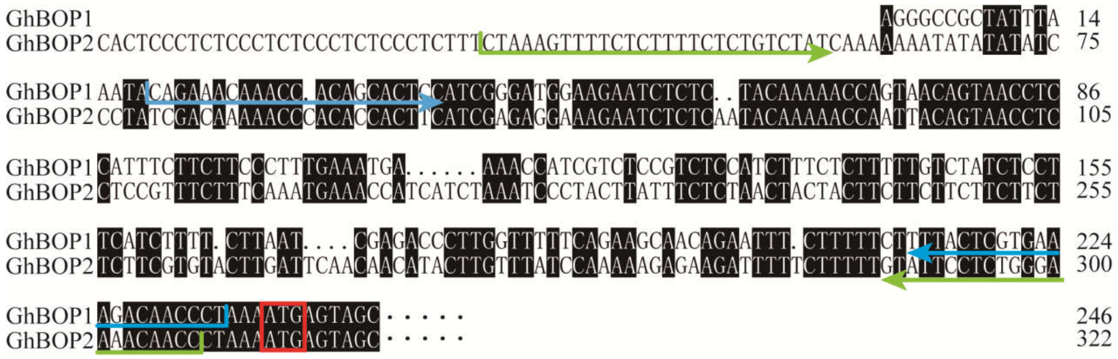

Supplementary Figure 11. Alignment of 5'UTR sequences between *GhBOP1* and *GhBOP2*. The opposite blue and green arrows indicate the specific primer sequences used for VIGS analysis of *GhBOP1* and *GhBOP2*, respectively. The red hollow frame indicates initiation codon.

Supplementary Figure 12

Figure 4B

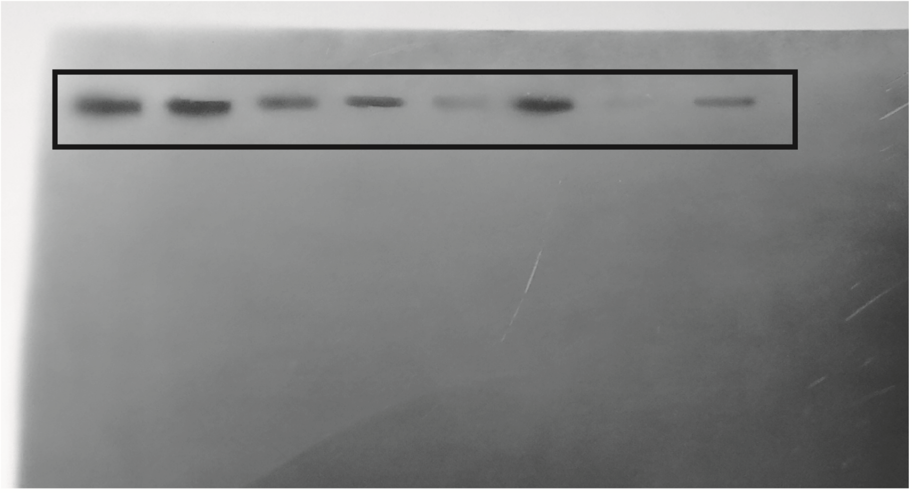

Figure 5A

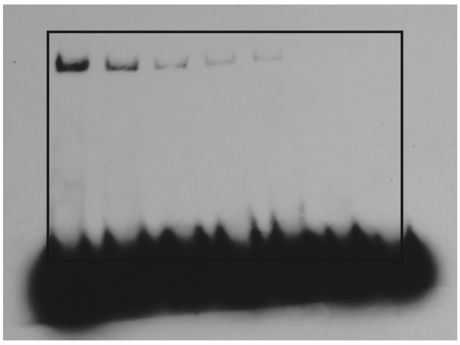

Figure 5B

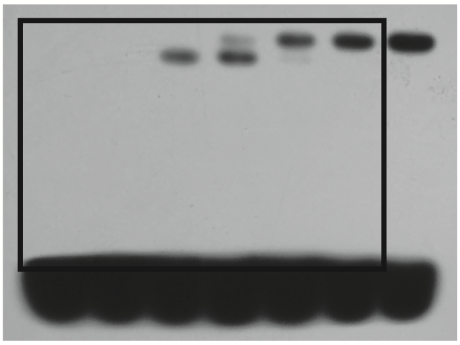

Supplementary Figure 12. **Uncropped blot and gel images.** The black rectangles shown the final cropped part of the image from main figures (figure 4 to 5).

Supplementary Table 1. Cis-element analysis of GhPR1 promoter

	motif name	motif sequence	motif site upstream of ATG	direction
PlantCARE_13222	ERE	ATTTTAAA	1228	+
PlantCARE_13222	MYB-like sequence	TAACCA	383	-
PlantCARE_13222	CGTCA-motif	CGTCA	846	+
PlantCARE_13222	AAGAA-motif	GGTAAAGAAA	1447	-
PlantCARE_13222	AAGAA-motif	GAAAGAA	347	+
PlantCARE_13222	P-box	CCTTTTG	1049	-
PlantCARE_13222	Unnamed__1	CGTGG	1068	+
PlantCARE_13222	GARE-motif	TCTGTTG	1004	-
PlantCARE_13222	I-box	TGATAATGT	1282	+
PlantCARE_13222	Box 4	ATTAAT	1382	+
PlantCARE_13222	Box 4	ATTAAT	1158	+
PlantCARE_13222	Box 4	ATTAAT	316	-
PlantCARE_13222	TGACG-motif	TGACG	846	-
PlantCARE_13222	AuxRR-core	GGTCCAT	403	+
PlantCARE_13222	TATC-box	TATCCCA	622	+
PlantCARE_13222	W box	TTGACC	786	+
PlantCARE_13222	F-box	CTATTCTCATT	1458	+
PlantCARE_13222	Unnamed__6	taTAAATATct	87	+
PlantCARE_13222	chs-CMA1a	TTACTTAA	1038	+
PlantCARE_13222	Sp1	GGGCGG	1175	+
PlantCARE_13222	WUN-motif	AAATTACT	728	-
PlantCARE_13222	WUN-motif	AAATTTCTT	589	-
PlantCARE_13222	AT~TATA-box	TATATA	1377	+
PlantCARE_13222	AT~TATA-box	TATATA	1375	+
PlantCARE_13222	AT~TATA-box	TATATA	1019	+
PlantCARE_13222	AT~TATA-box	TATATA	1017	+
PlantCARE_13222	AT~TATA-box	TATATAAA	771	-
PlantCARE_13222	AT~TATA-box	TATATA	769	-
PlantCARE_13222	AT~TATA-box	TATATA	767	-
PlantCARE_13222	AT~TATA-box	TATATA	765	-
PlantCARE_13222	AT~TATA-box	TATATA	376	-
PlantCARE_13222	AT~TATA-box	TATATAAA	191	-
PlantCARE_13222	AT~TATA-box	TATATA	189	-
PlantCARE_13222	CAAT-box	CAAT	1489	+
PlantCARE_13222	CAAT-box	CAAT	1484	+
PlantCARE_13222	CAAT-box	CCAAT	1406	-
PlantCARE_13222	CAAT-box	CAAAT	1394	+
PlantCARE_13222	CAAT-box	CAAAT	1331	-

PlantCARE_13222	CAAT-box	CAAT	1287	+
PlantCARE_13222	CAAT-box	CAAAT	1285	-
PlantCARE_13222	CAAT-box	CAAT	1057	-
PlantCARE_13222	CAAT-box	CAAT	1009	-
PlantCARE_13222	CAAT-box	CAAT	982	-
PlantCARE_13222	CAAT-box	CAAAT	880	+
PlantCARE_13222	CAAT-box	CAAAT	816	+
PlantCARE_13222	CAAT-box	CAAT	787	-
PlantCARE_13222	CAAT-box	CAAAT	753	+
PlantCARE_13222	CAAT-box	CAAAT	724	-
PlantCARE_13222	CAAT-box	CAAAT	699	-
PlantCARE_13222	CAAT-box	CAAT	692	+
PlantCARE_13222	CAAT-box	CAAT	558	+
PlantCARE_13222	CAAT-box	CAAT	540	-
PlantCARE_13222	CAAT-box	CAAAT	427	-
PlantCARE_13222	CAAT-box	CAAAT	422	-
PlantCARE_13222	CAAT-box	CAAAT	393	-
PlantCARE_13222	CAAT-box	CAAAT	388	+
PlantCARE_13222	CAAT-box	CCAAT	385	-
PlantCARE_13222	CAAT-box	CAAT	289	+
PlantCARE_13222	CAAT-box	CCAAT	235	+
PlantCARE_13222	CAAT-box	CAAT	234	+
PlantCARE_13222	CAAT-box	CAAAT	184	-
PlantCARE_13222	CAAT-box	CAAT	154	+
PlantCARE_13222	CAAT-box	CAAT	32	-
PlantCARE_13222	CAAT-box	CAAT	28	+
PlantCARE_13222	G-Box	CACGTT	1070	-
PlantCARE_13222	GATA-motif	GATAGGA	1064	+
PlantCARE_13222	O2-site	GATGATGTGG	913	+
PlantCARE_13222	GT1-motif	GGTTAAT	382	+
PlantCARE_13222	TATA-box	TATA	1496	+
PlantCARE_13222	TATA-box	ATTATA	1468	+
PlantCARE_13222	TATA-box	TATAA	1467	-
PlantCARE_13222	TATA-box	TATA	1466	+
PlantCARE_13222	TATA-box	ATATAT	1378	+
PlantCARE_13222	TATA-box	TATATA	1377	+
PlantCARE_13222	TATA-box	ATATAT	1376	+
PlantCARE_13222	TATA-box	TATATA	1375	+
PlantCARE_13222	TATA-box	ATATAT	1374	+
PlantCARE_13222	TATA-box	TATA	1373	+
PlantCARE_13222	TATA-box	TATAAATA	1364	-
PlantCARE_13222	TATA-box	TATAAAT	1363	-
PlantCARE_13222	TATA-box	TATAAA	1362	-
PlantCARE_13222	TATA-box	TATAA	1361	-

PlantCARE_13222	TATA-box	TATA	1360	+
PlantCARE_13222	TATA-box	ccTATAAAaa	1201	+
PlantCARE_13222	TATA-box	TATA	1199	+
PlantCARE_13222	TATA-box	ATATAA	1130	+
PlantCARE_13222	TATA-box	TATA	1129	+
PlantCARE_13222	TATA-box	TATAAA	1088	-
PlantCARE_13222	TATA-box	TATAA	1087	-
PlantCARE_13222	TATA-box	TATA	1086	+
PlantCARE_13222	TATA-box	ATTATA	1021	+
PlantCARE_13222	TATA-box	TATATAA	1020	-
PlantCARE_13222	TATA-box	TATATA	1019	+
PlantCARE_13222	TATA-box	ATATAT	1018	+
PlantCARE_13222	TATA-box	TATATA	1017	+
PlantCARE_13222	TATA-box	ATATAA	1016	+
PlantCARE_13222	TATA-box	TATA	1015	+
PlantCARE_13222	TATA-box	ATTATA	877	+
PlantCARE_13222	TATA-box	TATAA	876	-
PlantCARE_13222	TATA-box	TATA	875	-
PlantCARE_13222	TATA-box	TATAA	865	-
PlantCARE_13222	TATA-box	TATA	864	-
PlantCARE_13222	TATA-box	taTATAAAtc	773	-
PlantCARE_13222	TATA-box	TATAAAT	772	-
PlantCARE_13222	TATA-box	TATAAA	771	-
PlantCARE_13222	TATA-box	TATATAA	770	-
PlantCARE_13222	TATA-box	TATATA	769	-
PlantCARE_13222	TATA-box	ATATAT	768	-
PlantCARE_13222	TATA-box	TATATA	767	-
PlantCARE_13222	TATA-box	ATATAT	766	-
PlantCARE_13222	TATA-box	TATATA	765	-
PlantCARE_13222	TATA-box	ATATAA	764	+
PlantCARE_13222	TATA-box	TATA	763	-
PlantCARE_13222	TATA-box	TATAAATA	555	-
PlantCARE_13222	TATA-box	TATAAAT	554	-
PlantCARE_13222	TATA-box	TATAAA	553	-
PlantCARE_13222	TATA-box	TATAA	552	-
PlantCARE_13222	TATA-box	TATA	551	-
PlantCARE_13222	TATA-box	ATATAT	377	-
PlantCARE_13222	TATA-box	TATATA	376	-
PlantCARE_13222	TATA-box	ATATAT	375	-
PlantCARE_13222	TATA-box	TATA	374	-
PlantCARE_13222	TATA-box	ATTATA	302	+
PlantCARE_13222	TATA-box	TATAA	301	-
PlantCARE_13222	TATA-box	TATA	300	-
PlantCARE_13222	TATA-box	TATA	249	-

PlantCARE_13222	TATA-box	TATAAAA	192	-
PlantCARE_13222	TATA-box	TATAAA	191	-
PlantCARE_13222	TATA-box	TATATAA	190	-
PlantCARE_13222	TATA-box	TATATA	189	-
PlantCARE_13222	TATA-box	ATATAT	188	-
PlantCARE_13222	TATA-box	TATA	187	-
PlantCARE_13222	TATA-box	TATA	87	-
PlantCARE_13222	TATA-box	TATAAGAA	23	-
PlantCARE_13222	TATA-box	TATAA	20	-
PlantCARE_13222	TATA-box	TATA	19	-
PlantCARE_13222	ARE	AAACCA	224	+
PlantCARE_13222	MYB	CAACAG	1004	+
PlantCARE_13222	MYB	CAACAG	688	+
PlantCARE_13222	MYB	TAACCA	383	-
PlantCARE_13222	chs-CMA2a	TCACTTGA	1306	-
PlantCARE_13222	TCT-motif	TCTTAC	1098	-
PlantCARE_13222	Myb-binding site	CAACAG	1004	+
PlantCARE_13222	Myb-binding site	CAACAG	688	+
PlantCARE_13222	ABRE	ACGTG	1069	+
PlantCARE_13222	ABRE	TACGGTC	406	+

TGACG-element is highlight in red
