# Supplementary material for: Cotton plant defence against a fungal pathogen is enhanced by expanding BLADE-ON-PETIOLE1 expression beyond lateral-organ boundaries
Source: Commun Biol. 2019 Jun 21;2:238. doi: 10.1038/s42003-019-0468-5 (PMC6588604; doi:10.1038/s42003-019-0468-5)
Supplement: Supplementary file 2 — Description of Additional Supplementary Files [file 42003_2019_468_MOESM2_ESM.docx]

**Description of Additional Supplementary Files**

**File Name**: Supplementary Data1

**Description**: All source data involved in the main figures is listed in the Excel.
